# Phenotypic antibiotic susceptibility profile of clinical Enterobacteriaceae isolates from Kaduna State, northwest Nigeria

**DOI:** 10.1099/acmi.0.000747.v5

**Published:** 2024-06-20

**Authors:** Peace Amarachukwu Ezeh, Busayo Olalekan Olayinka, Rebecca Olajumoke Bolaji, Suleiman Ahmed Babangida, Ahmed Olowo-okere

**Affiliations:** 1 Department of Pharmaceutical Microbiology, Ahmadu Bello University, Zaria, Nigeria; 2 Department of Microbiology, Ahmadu Bello University, Zaria, Nigeria; 3 Department of Pharmaceutical Microbiology and Biotechnology, University of Abuja, FCT, Abuja, Nigeria

**Keywords:** antibiotic resistance, *Enterobacteriaceae*, multiple drug resistance

## Abstract

**Background.** The increasing resistance of clinical *Enterobacteriaceae* isolates to commonly prescribed antibiotics has been reported around the world. Data are generally lacking on the prevalence and antibiotic susceptibility profile of clinical *Enterobacteriaceae* isolates from Kaduna, northwest Nigeria. This study thus aimed to determine the diversity and antibiotic resistance profile of clinical *Enterobacteriaceae* isolates recovered from clinical specimens from patients admitted to two selected healthcare institutions in Kaduna.

**Methods.** This was a prospective cross-sectional study conducted between September and December 2021. Non-duplicate clinical bacterial isolates recovered from various specimens were collected and identified using rapid biochemical identification kits. The susceptibility of identified *Enterobacteriaceae* to various antibiotics and phenotypic detection of carbapenemase enzymes were thereafter determined. The data were analysed and visualized using R software version 4.3.1.

**Results.** Of the 500 bacterial isolates recovered from specimens collected for the purpose of clinical bacteriology diagnostics, 108 (21.6 %) were identified as *Enterobacteriaceae*, with *Pantoea agglomerans* (52, 48.1 %) and *Klebsiella oxytoca* (19, 17.6 %) being the most prevalent. The isolates exhibited high resistance to azithromycin (69 %) and ceftazidime (42 %), while exhibiting low resistance to amikacin (7 %) and imipenem (10 %). Among the carbapenem-resistant *Enterobacteriaceae* (CRE) isolates, a significant proportion (12/17, 70.6 %) tested positive for carbapenemase activity.

**Conclusion.** This study reports a high prevalence of multidrug-resistant *Enterobacteriaceae* in Kaduna, northwest Nigeria. The emergence of pathogenic *P. agglomerans* and an alarmingly high prevalence of carbapenemase-producing CRE were also observed. The presence of carbapenemase producers in an area with low carbapenem usage and resistance rates raises significant concerns. Continuous surveillance and robust antibiotic stewardship policies are imperative to preserve the efficacy of carbapenems in this region.

## Data Summary

Data generated from this study have been deposited on Figshare with the DOI https://doi.org/10.6084/m9.figshare.24849297.v2[1].

## Introduction

*Enterobacteriaceae* are a frequent source of various infections, ranging from mild through moderate to sometimes severe, life-threatening infections [2,4]. They have been included in the World Health Organization’s global list of priority pathogens that are resistant to antibiotics for prioritization in research and development (R&D) efforts for novel and efficient antibiotic treatments [5].

Recently, the World Health Organization identified antimicrobial resistance as one of the top 10 global public health threats [6]. In 2019, there were approximately 4.95 million (with a range of 3.62–6.57 million) deaths associated with bacterial antimicrobial resistance (AMR). This figure includes an estimated 1.27 million (with a 95 % uncertainty interval of 0.911–1.71 million) deaths specifically attributable to bacterial AMR. Moreover, the overall death rate related to resistance was highest in western sub-Saharan Africa, reaching 27.3 deaths per 100 000 (with a range of 20.9–35.3), and lowest in Australasia, at 6.5 deaths (with a range of 4.3–9.4) per 100 000 [7]. The World Bank projected that by 2050 as much as 3.8 % of the worldwide gross domestic product could be lost due to AMR [8]. Additionally, over 70 % of all pathogenic bacteria are said to exhibit resistance to at least one antibiotic that is commercially accessible [9].

Antimicrobial-resistant *Enterobacteriaceae* are a global public health threat, causing a range of hospital- and community-acquired infections [10]. Among all the challenges posed by bacterial resistance, Gram-negative pathogens are especially alarming, as they are increasingly developing resistance to almost all antibiotics suitable for treatment of the infections they cause [11]. According to the US National Healthcare Safety Network, Gram-negative bacteria accounted for 30 % of hospital-acquired infections and 70 % of infections in intensive care units. Among these bacteria, the family *Enterobacteriaceae* is the most frequently implicated [12]. According to a report from the World Health Organization (WHO), 45 % of deaths in Africa and South-East Asia are linked to multidrug-resistant (MDR) bacteria. In particular, *Klebsiella pneumoniae*’s resistance to third-generation cephalosporins was identified as a contributing factor to fatalities in Africa (77 %), the Eastern Mediterranean region (50 %), South-east Asia (81 %), and the Western Pacific region (72 %) [13]. *E. coli*, *Klebsiella pneumoniae*, and *Enterobacter* spp. have been reported as the most prevalent *Enterobacteriaceae* in Nigeria [14,15].

In developing nations such as Nigeria, there is a scarcity of comprehensive data regarding the surveillance of antibiotic-resistant *Enterobacteriaceae* and their resistance patterns in both hospital- and community-acquired infections [16]. The absence of evidence concerning the increase and spread of MDR *Enterobacteriaceae* between community and hospital settings poses a significant obstacle to raising awareness, implementing effective management strategies, and executing appropriate control measures against drug-resistant bacterial infections. The surveillance of the distribution of *Enterobacteriaceae* in the study population will provide knowledge of resistance phenotypes, and the magnitude of their prevalence within the period of study. It will further help in understanding the scale of emerging resistance and new resistance determinants, to inform policy direction on the use of antimicrobial agents. The aim of this research was to assess the antibiotic susceptibility patterns of clinical *Enterobacteriaceae* isolates obtained from two selected hospitals in Kaduna State, located in northwest Nigeria.

## Methods

### Ethical consideration

Ethical approval was requested and obtained from the hospital’s Ethics and Research Committee with the reference number ABUTHZ/HREC/W21/2020. Since this study does not involve direct collection of clinical specimens from patients, the requirement for obtaining consent from individual patients was waived by the committee.

### Study design and settings

This is a prospective cross-sectional study focusing on bacterial isolates obtained from various clinical specimens as part of bacteriological diagnostics at Ahmadu Bello University Teaching Hospital (ABUTH) and St Luke’s Hospital (SLH), both in Zaria, Kaduna State, Nigeria. The former hospital is a federal government-owned tertiary healthcare institution, while the latter is a privately operated hospital. Kaduna State is located in the northwest geopolitical zone of Nigeria and situated at approximately 10.52641°N latitude and 7.41667°E longitude. As at 2020, Kaduna State has an estimated population of 9.03 million, with Zaria local government being the second largest local government, having an estimated population of 601 300 people [17].

### Bacterial isolate collection and identification

Over a 3 month period between September and December 2021, a collection of 500 non-repetitive, consecutive clinical Gram-negative bacteria recovered from routine clinical specimens processed in the microbiology laboratories of the 2 hospitals were collected. Pre-designed data collection forms were used to collect socio-demographic data for the patients from whom the isolates were obtained. The specimens were acquired from individuals who sought medical care for various conditions and the samples were handled and processed in accordance with established guidelines in the laboratories for detection and isolation of microbial pathogens [18].

In brief, clean-catch midstream urine samples were collected and cultured on MacConkey agar, while wound samples were collected by swabbing the wound, and swabs were streaked onto MacConkey agar. The sputum samples collected were inoculated onto prepared blood and MacConkey agar plates. For blood culture, two sets of sterile draws (aerobic and anaerobic) from two separate venipuncture sites were drawn and the samples were then incubated in a blood culture incubator (BACTEC). The blood samples were then sub-cultured on blood agar and MacConkey agar. Stool samples were transported to the laboratory within 1–2 h of collection. One gram of each stool sample collected was inoculated into peptone water and then cultured from the peptone water into nutrient agar and MacConkey agar. Pus samples were obtained using sterile disposable cotton swabs and aspirates were collected in syringes. Following collection, the specimens were immediately transported and processed in the microbiology laboratory.

To collect throat samples, the oral cavity was opened widely, with adequate illumination, and the tongue depressed with a sterile wooden specular, and then the sterile swab stick was rubbed firmly on the surfaces of the infected regions, avoiding contact with the tongue. For endocervical and vaginal swabs, using a speculum and under direct visualization of the cervix, the endocervix was swabbed using a sterile cotton swab by inserting the cotton swab 20–30 mm into the cervical canal and rotating the swab gently through 360° clockwise against the endocervical wall while sterile cotton swabs were inserted and rotated 360° onto the posterior vaginal fornix under direct vision. Each of the swabs collected was inserted back into the tube from which it had been taken, and then transported to the laboratory. Patients with discharge from one or both ears had ear swabs taken by an otorhinolaryngologist for microbiological analysis. Blood agar and MacConkey agar media were used to inoculate the specimens from the swabs using the streaking method. All samples were cultured at 37 °C for 24–48 h.

Semen specimens were obtained through masturbation (non-coital) following a 3–5 day period of sexual abstinence and refraining from antibiotics. The collected samples were placed in appropriately labelled sterile containers, securely sealed, and maintained at body temperature (37 °C). Timely transportation to the laboratory within 15 min of production was ensured. Subsequently, a loopful of semen samples was promptly collected and inoculated onto both blood agar and MacConkey agar. Bacterial isolates that grew on each culture media were then collected for further analysis to screen for *Enterobacteriaceae*.

### Screening for *Enterobacteriaceae*

To screen for *Enterobacteriaceae*, the collected bacterial isolates were purified by repeated sub-culturing on MacConkey agar. A colony of the isolate was picked using a loop and streaked onto plates containing the differential media aseptically as described by Sanders [19]. Plates were inverted and incubated overnight at 35 °C. Isolates that grew on the MacConkey agar were screened using Gram staining and catalase tests. The catalase-positive, Gram-negative isolates were further identified using a rapid micro-substrate biochemical-based identification system, the Microbact Gram-negative ID kit (Microbact GNB 12A kit, Thermo Scientific Oxoid Ltd, UK) following the manufacturer’s instructions. From an 18–24 h culture on MacConkey agar, one to three isolated colonies were selected and emulsified in 2.5 ml of sterile saline solution. This mixture was blended thoroughly to create a uniform suspension. While the plate was positioned in the holding tray, approximately 100 µl (four drops) of the bacterial suspension were dispensed onto the plates using a sterile Pasteur pipette. The substrates marked on the holding tray (wells 1, 2, and 3) were then covered with sterile mineral oil using a dropper bottle. The inoculated rows were sealed again with an adhesive seal, and the specimen identification number was inscribed on the end tag using a marker pen. Subsequently, the prepared plates were incubated at 35±2 °C for a duration of 18–24 h. Positive reactions were defined using the colour chart and the results were recorded. The Microbact computer-aided identification package was used for identification of the isolates to species level.

### Determination of antimicrobial susceptibility of isolates

The *Enterobacteriaceae* isolates that were identified were tested for antibiotic susceptibility using the modified Kirby–Bauer disc diffusion method on Mueller–Hinton agar plates, following the guidelines outlined in the Clinical and Laboratory Standards Institute (CLSI) guideline of 2020 [20] . The antibiotic discs utilized in this investigation included ceftazidime (30 µg), imipenem (10 µg), azithromycin (15 µg), meropenem (10 µg), ciprofloxacin (5 µg), chloramphenicol (30 µg), levofloxacin (5 µg), amikacin (30 µg), gentamicin (10 µg), cefotaxime (30 µg), and trimethoprim/sulfamethoxazole (25 µg), all sourced from Oxoid Ltd in the UK. A suspension of overnight growth of each isolate on nutrient agar plates was standardized by comparing the turbidity with 0.5 McFarland standards corresponding to approximately 1.5×10^8^ c.f.u. ml^−1^. A suspension of the isolates was inoculated on Mueller-Hinton agar plate using a sterile swab. The swab was streaked evenly over the surface of the medium to ensure confluent growth. The surface of the agar was allowed to dry for 5 min and the antibiotic discs were placed on the surface of the agar using sterile forceps. After 30 min of applying the discs, plates were inverted and incubated at 37 °C for 18 h. After overnight incubation, plates were examined and the diameter of each zone of growth inhibition around the discs were measured in mm, using a ruler on the underside of the plate. The diameter of the zone of inhibition was measured and interpreted according to CLSI interpretive criteria and the isolates were categorized as susceptibile or resistant. The isolates were thereafter categorized into the various multidrug-resistant (MDR) types according to the consensus definition published in [21].

### Phenotypic detection of carbapenemase production

The modified carbapenem inactivation method (mCIM) was conducted in accordance with CLSI guidelines [20] for isolates that were resistant to carbapenem using a standard 10 µg meropenem disc. Briefly, the test was performed by first inoculating 1 µl loopful of the test bacteria into a test tube containing tryptic soy broth. After a 10 s vortexing of the test tube, a 10 µg meropenem disc was added using sterile forceps, and the culture was incubated at 37 °C for 4 h. Thereafter, the disc was removed and transferred to the surface of a Mueller–Hinton agar plate previously swabbed with a standardized 0.5 McFarland suspension of *E. coli* ATCC 25922. The plates were inverted and incubated at 37 °C for 18–24 h. Following incubation, the zone of inhibition around the meropenem disc was measured and the results were interpreted as follows: carbapenemase-positive if the diameter zone of inhibition around the meropenem disc was 6–15 mm or colonies were present within a 16–18 mm zone and carbapenemase =-negative if the diameter zone of inhibition was ≥19 mm.

### Data analysis

Descriptive analysis was carried out by calculating frequencies and percentages to summarize the demographic and clinical data for the patients. The results were presented using tables and charts. Statistical analysis and visualization were performed using R version 4.3.1 and the RStudio interface. The overall resistance of bacterial strains was presented using a horizontal bar chart, while the individual resistance profile of each strain to various antibiotics was depicted via a heatmap. The heatmap was generated using ggplot2 and reshape2 packages in RStudio. In both visualisations, colour-coded bars and gradients represented distinct resistance categories. Increasing colour intensity denotes an increasing proportion of resistant isolates. The resistance levels was defined as low, moderate, and high, categorized respectively for values falling below 30 %, between 30 and 60 %, and exceeding 60 %.

## Results

### Patient characteristics

During the sample collection period, a total of 500 bacterial isolates comprising 340 (68 %) and 160 (32 %) were collected from ABUTH and SLH. The isolates were recovered from 286 (57.2 %) female and 214 (42.8 %) male patients, respectively. The vast majority of the isolates (*n*=334, 66.8 %) were recovered from patients aged 18–35 years. The distribution of isolates according to specimen sources showed that most of the bacteria (*n*=137, 27.4 %) were obtained from urine samples. Additional sources included stool samples (114; 22.8 %) and wound swabs (60; 12 %) (Table 1).

**Table 1.** Sociodemographic characteristics of patients involved in the study

Characteristics	Frequency (percentage)
**Gender**	
Female	287 (57.4 %)
Male	213 (42.6 %)
**Age categories**	
Below 18 years	32 (6.4 %)
18–35 years	334 (66.8 %)
36–55 years	108 (21.6 %)
Above 55 years	26 (5.2 %)
**Isolate source**	
Urine	137 (27.4 %)
Stool	114 (22.8 %)
Endocervical and vaginal swabs	105 (21 %)
Wound	60 (12 %)
Sputum	58 (11.6 %)
Pus	10 (2 %)
Blood	9 (1.8 %)
Throat	4 (0.8 %)
Ear	2 (0.4 %)
Seminal	1 (0.2 %)
Total	500 (100 %)

### Identification of isolates

The prevalence of *Enterobacteriaceae* from this study was 21.6 % (108/500). The *Enterobacteriaceae* isolated from this study include *Pantoea agglomerans* (52, 48.1 %), *Klebsiella oxytoca* (19, 17.6 %)*, E. coli* (11, 10.2 %), *Providencia rettgeri* (6, 5.6 %)*, Proteus vulgaris* (4, 3.7 %)*, Proteus mirabilis* (3, 2.8 %)*, Providencia stuartii* (3, 2.8 %), *Serratia rubidaea* (3, 2.8 %)*, Klebsiella ozaenae* (2, 1.9 %)*, Salmonella* spp. (2, 1.9 %)*, Citrobacter freundii* (1, 0.9 %)*, Yersinia enterocolitica* (1, 0.9 %), and *Morganella morganii* (1, 0.9 %) (Table 2). The *Enterobacteriaceae* isolates were recovered from 61 (56 %) female and 47 (44 %) male patients, respectively. The vast majority of the *Enterobacteriaceae* (*n*=59, 55 %) were recovered from patients aged 18–35 years. The distribution of bacteria isolated from hospitals indicates notable variations. *Klebsiella* spp. (29.7 %), *Pantoea* spp. (42.2 %), and *E. coli* (9.4 %) exhibited higher prevalence in ABUTH compared to SLH. Furthermore, when considering gender-specific trends, *Pantoea* spp. (56.8 %) and *Salmonella* spp. were notably more prevalent in samples from women compared to men, while *E. coli* (9.4 %), *Klebsiella* spp. (14.8 %), and *Morganella* spp. (1.6 %) showed higher occurrence in samples from men. Additionally, analysing sample sources revealed distinct bacterial preferences. *Pantoea* spp. emerged as the most frequently isolated bacteria across multiple sample types, including sputum (53.8 %), stool (35 %), urine (59.5 %), and wound (43.8 %) samples, indicating that it is widespread in clinical specimens (Table 2).

**Table 2.** Distribution of *Enterobacteriaceae* isolates across various determinants

Determinants		*Citrobacter* spp.	*E. coli*	*Klebsiella* spp.	*Morganella* spp.	*Pantoea* spp.	*Proteus* spp.	*Providencia* spp.	*Salmonella* spp.	*Serratia* spp.	*Yersinia* spp.	Total
**Hospital**	ABUTH Shika	1 (1.6 %)	6 (9.4 %)	19 (29.7 %)	0 (0.0 %)	27 (42.2 %)	2 (3.1 %)	5 (7.8 %)	0 (0.0 %)	3 (4.7 %)	1 (1.6 %)	64 (59.3 %)
	ST Luke’s Wusasa	0 (0.0 %)	5 (11.4 %)	2 (4.5 %)	1 (2.3 %)	25 (56.8 %)	5 (11.4 %)	4 (9.1 %)	2 (4.5 %)	0 (0.0 %)	0 (0.0 %)	44 (40.7 %)
**Gender**	Female	1 (1.6 %)	4 (6.6 %)	9 (14.8 %)	1 (1.6 %)	35 (57.4 %)	3 (4.9 %)	5 (8.2 %)	2 (3.3 %)	1 (1.6 %)	0 (0.0 %)	61(56%)
	Male	0 (0.0 %)	7 (14.9 %)	12 (25.5 %)	0 (0.0 %)	17 (36.2 %)	4 (8.5 %)	4 (8.5 %)	0 (0.0 %)	2 (4.3 %)	1 (2.1 %)	47 (44 %)
**Age**	Below 18	0 (0.0 %)	1 (12.5 %)	2 (25.0 %)	0 (0.0 %)	2 (25.0 %)	1 (12.5 %)	1 (12.5 %)	1 (12.5 %)	0 (0.0 %)	0 (0.0 %)	8 (7 %)
	18–35	1 (1.7 %)	5 (8.5 %)	9 (15.3 %)	0 (0.0 %)	33 (55.9 %)	4 (6.8 %)	4 (6.8 %)	0 (0.0 %)	2 (3.4 %)	1 (1.7 %)	59 (55 %)
	36–55	0 (0.0 %)	4 (12.5 %)	8 (25.0 %)	1 (3.1 %)	13 (40.6 %)	1 (3.1 %)	3 (9.4 %)	1 (3.1 %)	1 (3.1 %)	0 (0.0 %)	32 (30 %)
	Above 55	0 (0.0 %)	1 (11.1 %)	2 (22.2 %)	0 (0.0 %)	4 (44.4 %)	1 (11.1 %)	1 (11.1 %)	0 (0.0 %)	0 (0.0 %)	0 (0.0 %)	9 (8 %)
**Isolate source**	Cervical swab	0 (0.0 %)	3 (15.8 %)	6 (31.6 %)	0 (0.0 %)	8 (42.1 %)	0 (0.0 %)	1 (5.3 %)	0 (0.0 %)	1 (5.3 %)	0 (0.0 %)	19 (17.6 %)
	Pus	0 (0.0 %)	1 (33.3 %)	1 (33.3 %)	0 (0.0 %)	1 (33.3 %)	0 (0.0 %)	0 (0.0 %)	0 (0.0 %)	0 (0.0 %)	0 (0.0 %)	3 (3 %)
	Sputum	0 (0.0 %)	0 (0.0 %)	2 (15.4 %)	0 (0.0 %)	7 (53.8 %)	2 (15.4 %)	2 (15.4 %)	0 (0.0 %)	0 (0.0 %)	0 (0.0 %)	13 (12 %)
	Stool	0 (0.0 %)	2 (10.0 %)	6 (30.0 %)	1 (5.0 %)	7 (35.0 %)	1 (5.0 %)	1 (5.0 %)	1 (5.0 %)	1 (5.0 %)	0 (0.0 %)	20 (19 %)
	Urine	0 (0.0 %)	5 (13.5 %)	2 (5.4 %)	0 (0.0 %)	22 (59.5 %)	3 (8.1 %)	3 (8.1 %)	1 (2.7 %)	0 (0.0 %)	1 (2.7 %)	37 (34 %)
	Wound	1 (6.3 %)	0 (0.0 %)	4 (25.0 %)	0 (0.0 %)	7 (43.8 %)	1 (6.3 %)	2 (12.5 %)	0 (0.0 %)	1 (6.3 %)	0 (0.0 %)	16 (15 %)
	**Total**											**108**(100 %)

### Antimicrobial susceptibility testing for *Enterobacteriaceae* isolates

**Fig. 1.**
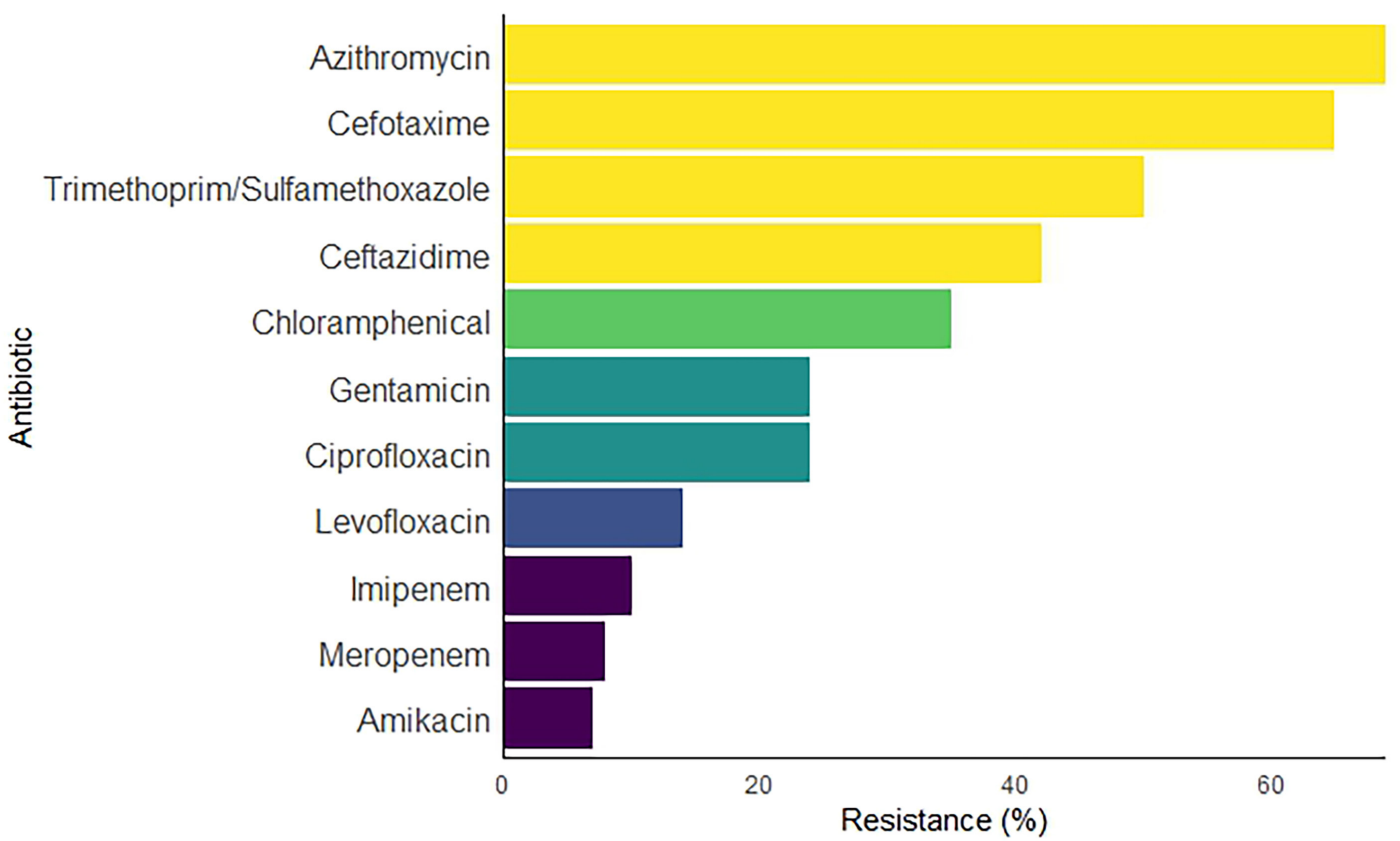
Overall antibiotic resistance pattern for the isolates. The length of the bars represents the overall level of resistance of the isolates to various antibiotics. The yellow bars denote high resistance exhibited by the isolates against specific antibiotics, while shades of green and blue represent moderate and low resistance, respectively.

**Fig. 2.**
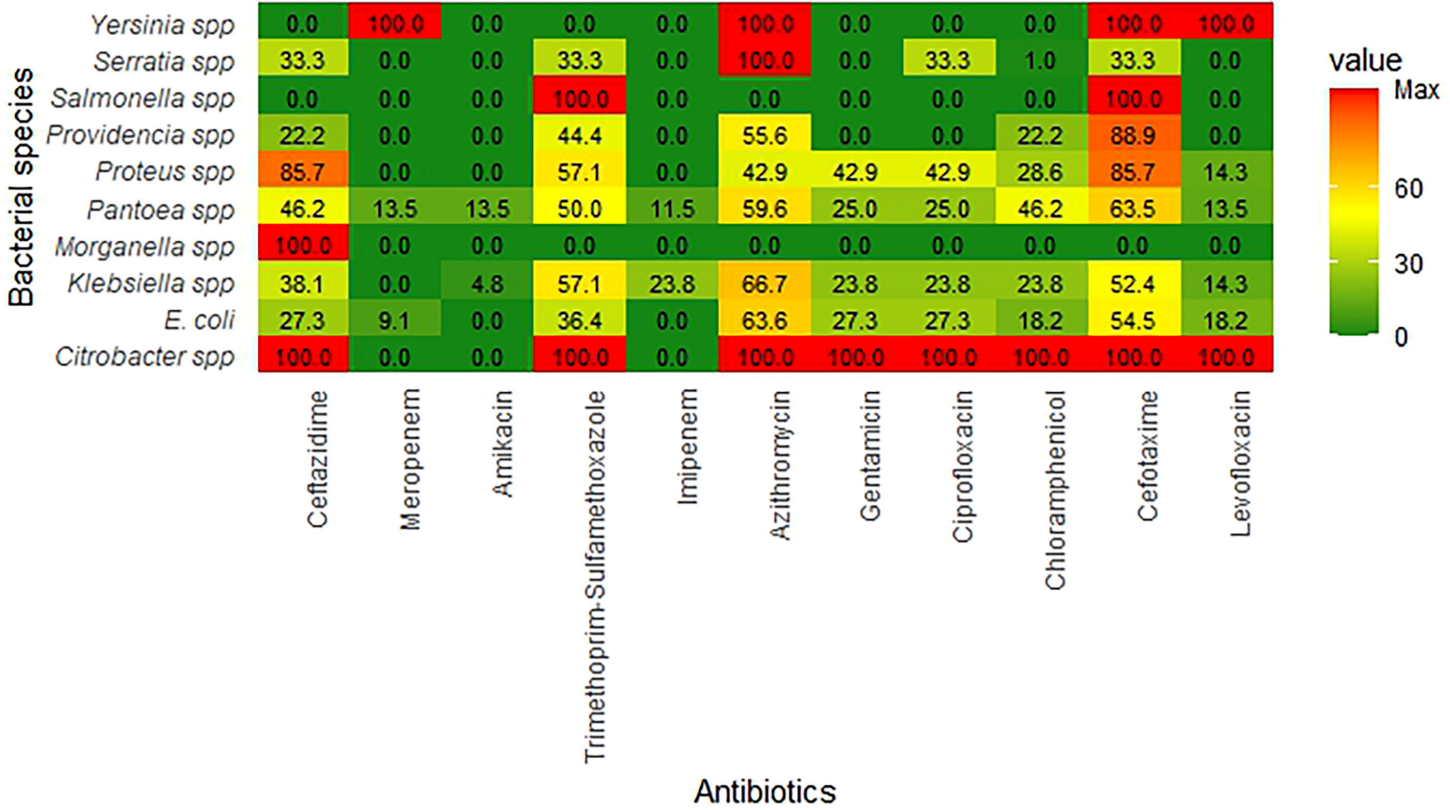
Antibiotic resistance patterns across *Enterobactericaeae* species. The heatmap illustrates the varying levels of antibiotic resistance across different *Enterobacteriaceae* species. Colour intensity represents the resistance levels, with red shades indicating higher resistance (>60 %), yellow shades denoting moderate resistance (30–60 %) and green shades showing low resistance (<30 %). Increasing colour intensity denotes an increasing proportion of resistant isolates.

Overall, the isolated *Enterobacteriaceae* were highly resistant to azithromycin (69 %) and cefotaxime (65 %). Moderate resistance was observed with trimethoprim/sulfamethoxazole (50 %), ceftazidime (42 %), and chloramphenicol (35 %), while low resistance was observed with amikacin (7 %), meropenem (8 %), and imipenem (10 %) (Fig. 1). The resistance pattern for each of member of *Enterobacteriaceae* isolated in this study is presented in Fig. 2.

**Fig. 3.**
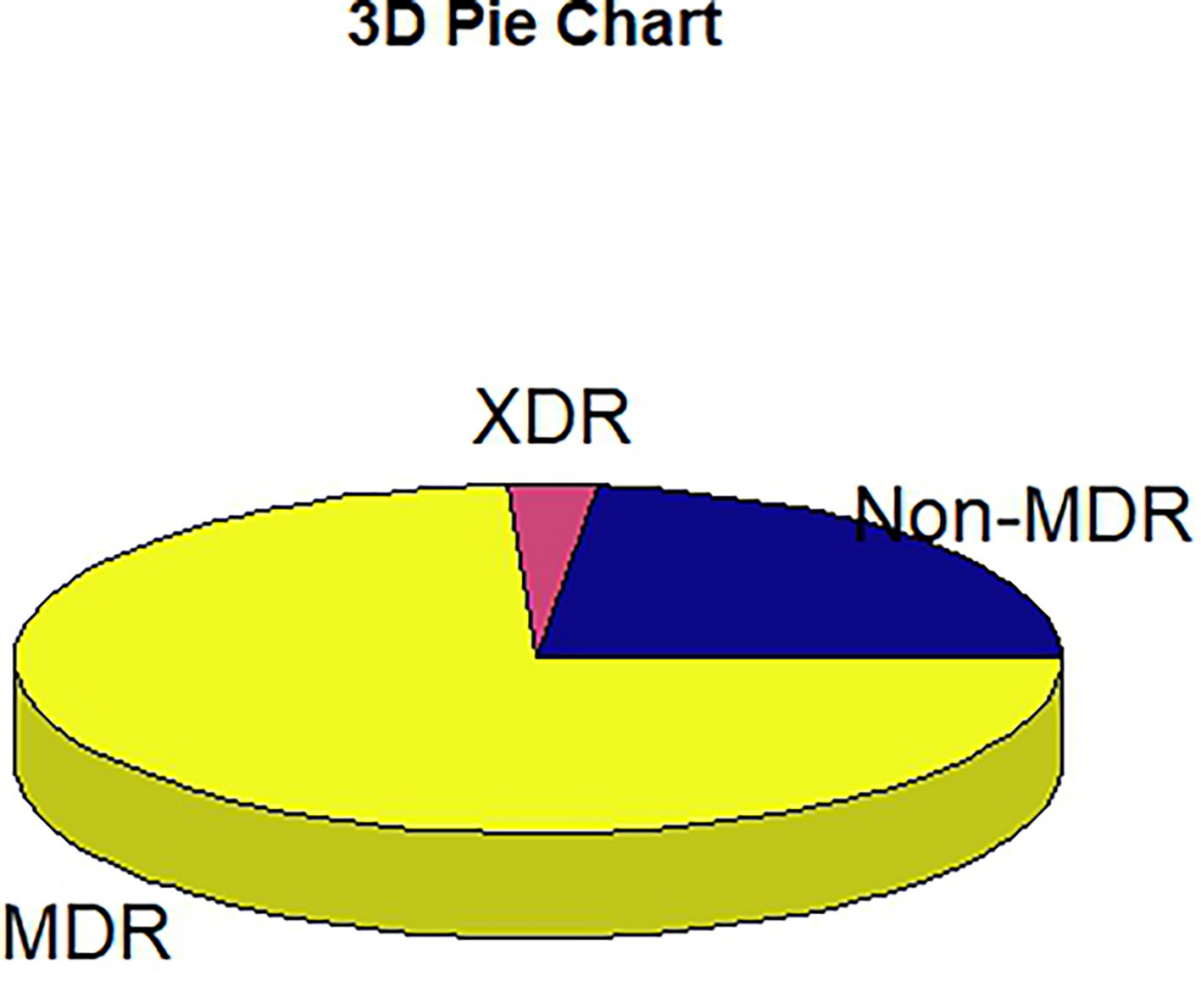
MDR phenotypes among the isolates. MDR, multidrug-resistant; XDR, extensively drug-resistant; non-MDR, non-multidrug-resistant.

This study revealed a high prevalence of multidrug resistance (73 %, 79/108), with 3/108 (3 %) showing extensive drug resistance and 26/108 (24 %) showing non-multidrug resistance (Fig. 3). Within the isolates, 17/108 (15.7 %) demonstrated non-susceptibility to carbapenems. This included *P. agglomerans* (10/17), *Y. enterocolitica* (1/17), *E. coli* (1/17), and *K. oxytoca* (5/17). Of the 17 isolates that showed resistance to carbapenem, the modified carbapenem inactivation test indicated positive results for 12/17 (70.6 %) carbapenem-resistant *Enterobacteriaceae* (CRE), suggesting potential carbapenemase expression in these isolates.

## Discussion

The rise of resistance within the clinical family *Enterobacteriaceae* has become a cause of concern worldwide. The diversity of bacteria isolated from clinical specimens in this study is in line with the reports of several other researchers [22]. The *Enterobacteriaceae* comprise a diverse array of bacteria with varying capacities for causing disease, ranging from symbiotic commensal microbiota with beneficial roles, to opportunistic pathogens that pose significant risks of morbidity and mortality, particularly to immunocompromised individuals, and primary pathogens capable of initiating illness even in individuals without underlying health conditions [23].

In line with the results of previous studies, this study observed a higher incidence of *Enterobacteriaceae* isolation in females compared to males [24,25]. Various factors, such as anatomical differences, hygiene practices, and immune status, may contribute to a heightened susceptibility of females to *Enterobacteriaceae* infections compared to their male counterparts, in addition to potentially non-random selection of isolates [26]. It has also been suggested that greater utilization of healthcare services by females, likely due to their greater healthcare needs compared to males, may further expose them to colonization and infection by healthcare-associated pathogens, notably *Enterobacteriaceae* [27,28]. Nevertheless, further investigation is warranted to thoroughly assess the relationship between gender and the prevalence of *Enterobacteriaceae* infections.

In the present study, *Enterobacteriaceae* isolates were predominantly recovered from patients aged 18 to 55 years. Similar findings have been reported in other studies [29]. Individuals aged 16 to 45 years, often more sexually active, have been reported to show increased susceptibility to bacterial infections [29].

This study also found a higher incidence of *Enterobacteriaceae* in urine samples. Similar trends have been observed in other studies [30]. While *Enterobacteriaceae* have been isolated in various clinical specimens, they are more frequently detected in urine samples, primarily due to their propensity to contaminate the urinary tract, particularly in women, where the proximity of the anus to the urethral opening increases the likelihood of migration and contamination of urinary tract with gut microbiota [31].

Interestingly, in this study, *Pantoea agglomerans* (formerly *Enterobacter agglomerans*) was the most prevalent bacterial species isolated from the collected specimens. A similarly high prevalence of *P. agglomerans* of 32 and 53 % has been reported previously [32,33]. Generally, *P. agglomerans* is an opportunistic pathogen in humans, mostly isolated from immuno-compromised patients or as a healthcare-associated pathogen in hospitals [34]. While clinical infections caused by *P. agglomerans* have been reported in the Nigerian literature, this is the first report of its predominance among clinical bacterial isolates in northwest Nigeria [35]. Clinical infection caused by a related species, *Pantoea dispersa*, has also been reported among patients admitted in a tertiary healthcare facility in Sokoto, northwest Nigeria [36]. The sudden surge in the prevalence of *P. agglomerans* among clinical pathogens in recent years may suggest a change in the pathogenicity of *Pantoea* spp. or a decreased level of immunity of patients in our hospitals. There is thus a need for further studies to understand the reasons for this.

Consistent with the findings of Yablon *et al.* [37], *Pantoea* spp. were found to be more prevalent in samples obtained from females than males. Conversely, other studies have documented a higher prevalence of *P. agglomerans* among males [38]. This variation may stem from regional differences or varying hygiene practices across those regions. While previous research consistently reports higher *E. coli* and *Klebsiella* spp. isolation in females, in line with the findings of Ngwai *et al.* [39] and Wang *et al.* [40], our study found these bacterial species to be more prevalent in males. Further investigations with larger sample sizes are needed to elucidate the underlying reasons for this variation.

The antibiotic resistance results revealed a high MDR phenotype among the isolates. The high resistance rates of the isolates to azithromycin (69 %) and cefotaxime (65 %) are comparable to those in other studies [41]. The high resistance to azithromycin observed in this study is not surprising since the antibiotic is known to possess limited activity against Gram-negative bacteria. The finding of high resistance to the third-generation cephalosporin, cefotaxime, is in line with current pattern of resistance in Nigeria. ESBL and carbapenemase enzyme-mediated resistance to third-generation cephalosporins have been reported in almost all states of the federation [42]. The low resistance rates of the isolates to amikacin (7 %), meropenem (8 %), imipenem (10 %), and levofloxacin (14 %) are comparable to those in other studies [36].

The high MDR phenotypes observed in this study is in agreement with findings from around the country. For example, in northwest Nigeria, 88.9 % has been reported [42], with 68.2 % in the south-south [43] and 56.2 % in the southwest [44]. The high prevalence of MDR might be due to a combination of microbial characteristics, increased consumption of antibiotics and the attendant resistance, and transmission of resistant isolates within the hospital environment as a result of insufficient enforcement of hospital hygiene protocols and infection control measures [45]. Although data on antibiotic consumption in the study area are limited, some studies have established a high level of use in agricultural practice within the study area [46]. Furthermore, overcrowding of immuno-compromised patients in the hospitals may facilitate nosocomial transmission of resistant bacteria among the patients [47].

The prevalence of carbapenem-resistant *Enterobacteriaceae* (CRE) ranges from 2–60 % in Africa, depending on the geographical location. In this study, low resistance was recorded to imipenem (10%) and meropenem (8%). This is similar to a study in Lagos, Nigeria, which reported 15.2 % prevalence of *Enterobacteriaceae* resistant to carbapenem [48]. Other studies also reported similar rates of resistance to carbapenems to those seen in this study. Resistance rates of 5.2 and 6.5 %, respectively, have been reported [36,49]. However, the report from this study is higher than the 2.6 % resistance recorded in Ghana in [50] and lower than the 28 % resistance reported in [51]. The low usage of carbapenems in Nigeria as a reserve antibiotic may be responsible for the usually low resistance recorded in this study and other similar studies in the country. However, there is a need for continuous surveillance in hospital settings.

Phenotypic detection of carbapenemase activity in, 70.6 % (*n*=12) of the CRE is comparable to the findings of a study by Pyakurel *et al*. [52], who reported a prevalence of 40 % for carbapenemase-producing *K. pneumoniae* in at a tertiary care hospital in Nepal. In Nigeria, 32.6 % carbapenemase-producing CRE has also been reported [51]. This is a worrying development, as the efficacy of one of the last-resort antibiotics in Nigeria is under threat. There is thus urgent need to institute an antibiotic stewardship policy to preserve the efficacy of this hitherto highly effective antibiotic.

The prospective design of the study, coupled with the collection of samples from multiple centres, including both federal and privately operated hospitals, significantly bolstered the robustness of our findings. It also enabled the inclusion of diverse patient populations, enhancing the generalizability of our results. However, the study was constrained by the unavailability of data on specific disease conditions. This limitation hinders our ability to correlate *Enterobacteriaceae* infections with underlying clinical conditions. Our inability to characterize the isolates molecularly was another limitation. This study, however, provides valuable insights into the diversity and antibiotic susceptibility profile of clinical *Enterobacteriaceae* within the study population, enriching our understanding of these microbial pathogens and informing targeted intervention strategies.

## Conclusion

This study reported a strikingly high prevalence of MDR *Enterobacteriaceae* in Kaduna, northwest Nigeria. It also brought to fore the growing pathogenic significance of *P. agglomerans* and the alarming prevalence of carbapenemase-producing CRE. This high occurrence of carbapenemase-producing *Enterobacteriaceae* in a region characterized by limited carbapenem use and resistance rates is a matter of grave concern. The findings underscore the need for continuous surveillance to monitor resistance patterns closely and implementation of antibiotic stewardship policies to safeguard the efficacy of carbapenem antibiotics.

## Abbreviations

ABUTH: Ahmadu Bello University Teaching Hospital
AMR: antimicrobial resistance
CLSI: Clinical and Laboratory Standards Institute
CRE: carbapenem-resistant *Enterobacteriaceae*
mCIM: modified carbapenem inactivation method
MDR: multi-drug resistant
Non MDR: non-multidrug resistant
SLH: St Luke's Hospital
XDR: extensively drug resistant
